# A Case of 17q21.31 Microduplication and 7q31.33 Microdeletion, Associated with Developmental Delay, Microcephaly, and Mild Dysmorphic Features

**DOI:** 10.1155/2014/658570

**Published:** 2014-02-04

**Authors:** Adrian Mc Cormack, Juliet Taylor, Leah Te Weehi, Donald R. Love, Alice M. George

**Affiliations:** ^1^Diagnostic Genetics, LabPlus, Auckland City Hospital, P.O. Box 110031, Auckland 1148, New Zealand; ^2^Genetic Health Service New Zealand-Northern Hub, Auckland City Hospital, Private Bag 92024, Auckland 1142, New Zealand; ^3^School of Biological Sciences, University of Auckland, Private Bag 92019, Auckland 1142, New Zealand

## Abstract

Concurrent cryptic microdeletion and microduplication syndromes have recently started to reveal themselves with the advent of microarray technology. Analysis has shown that low-copy repeats (LCRs) have allowed chromosome regions throughout the genome to become hotspots for nonallelic homologous recombination to take place. Here, we report a case of a 7.5-year-old girl who manifests microcephaly, developmental delay, and mild dysmorphic features. Microarray analysis identified a microduplication in chromosome 17q21.31, which encompasses the *CRHR1, MAPT,* and *KANSL1* genes, as well as a microdeletion in chromosome 7q31.33 that is localised within the *GRM8* gene. To our knowledge this is one of only a few cases of 17q21.31 microduplication. The clinical phenotype of patients with this microduplication is milder than of those carrying the reciprocal microdeletions, and suggests that the lower incidence of the former compared to the latter may be due to underascertainment.

## 1. Introduction

Since the advent of microarray technology considerable progress has been made in identifying small scale chromosome imbalances. The existence of colocalized microdeletion and microduplication syndrome sites has come to the fore in the recent years and a significant number of new microduplication syndromes have emerged such as 17p11.2 [1] and 22q11.21 [2]. These syndromes, like the corresponding microdeletion syndromes at these locations, appear to be driven by nonallelic homologous recombination (NAHR) involving low-copy repeats (LCRs or segmental duplications) [3–8]. LCRs are DNA fragments greater than 1 Kb in size, have 90% DNA sequence homology, and are thought to account for approximately 3–10% of the total genome.

The *MAPT* gene located on chromosome 17q21.31 is flanked by LCRs and two extended haplotypes, designated H1 and H2, have been identified [9, 10]. The H2 haplotype is a 900 kb inversion polymorphism that has been reported as the likely ancestral state and which has a tendency to undergo recombination [11] leading to the 17q21.31 microdeletion syndrome. This syndrome has been well characterised and appears to be caused by haploinsufficiency of at least one gene, *KANSL*, within the deleted region [12, 13]. The more common H1 haplotype appears to be overrepresented in patients manifesting progressive supranuclear palsy [14].

Here, we report a 7.5-year-old girl with a 647 kb duplication involving interstitial chromosome region 17q21.31 as well as a 232 kb heterozygous interstitial deletion involving chromosome region 7q31.33. We review this case in conjunction with other 17q21.31 microduplication cases described by Kirchhoff et al. [15], Kitsiou-Tzeli et al. [16], and Grisart et al. [17]. Our case shares some common phenotypic features with previously reported patients, including developmental delay, microcephaly, and mild dysmorphisms, which are milder than those identified in patients with the 17q21.31 microdeletion syndrome.

## 2. Clinical Report

The proband was the first born girl to nonconsanguineous Iraqi and Afghani parents. Family history on the mother's side was unremarkable. The father reportedly struggled at school and it has been suggested that he may have been microcephalic. The child was delivered at 38-week gestation via induction as there were concerns about IUGR. She was born in good condition and did not require resuscitation. Birth weight was 2490 g (3rd to 10th centile) and there were no other antenatal complications. Milestones were appropriate for age, walking at 15 months but always on her toes. She was initially referred to a child development service as she was falling a lot when walking and was prescribed orthoses. At the age of 2 years and 7 months she was further referred to a pediatric clinic because of her gait pattern. It was noted that she still had difficulty with toe walking, lack balance, and control when walking along slopes and stairs and was still learning how to do other developmental skills such as climbing, jumping, and pushing a bike. There were no obvious dysmorphic features. Her weight was 15.55 kg (90th centile), height was 92 cm (25th–50th centile), and head circumference was 46 cm (25th centile). A follow-up visit at 3 years of age showed that the idiopathic toe walking had resolved and that gross motor skills were continuing to develop. At the age of 5.5 years, she was found to be functioning at a level well below chronological age and was noted to be microcephalic (head circumference of 47 cm; 2nd centile) as well as developmentally delayed. Dysmorphic features were not observed at this visit, but some mild autistic traits were seen.

Developmental delay was further confirmed 2 years later, functioning at 4-5-years old level both academically and for fine motor skills. She required significant assistance with activities of daily life. It was also noticed at this visit that she had some dysmorphic features with possible almond shaped eyes and small hands.

## 3. Cytogenetic and Molecular Studies

**Figure 1:**
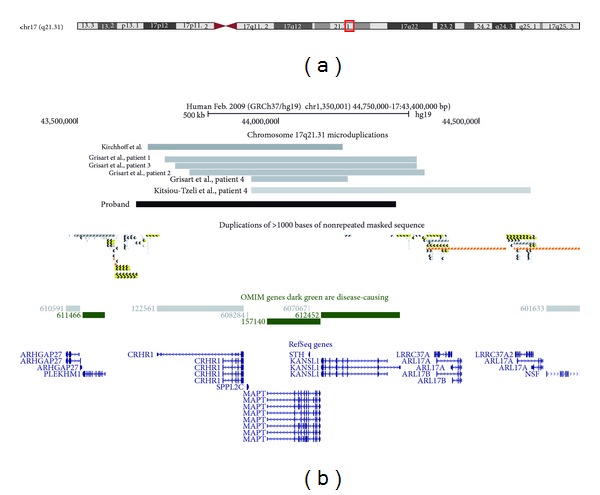
Schematic of the chromosome 17 region containing the microduplication. Panel A shows an ideogram of chromosome 17, together with the region encompassing the microduplications. Panel B shows the location and extent of the duplications detected in the proband reported here, other cases reported in the literature, LCRs (segmental duplications), and OMIM and Refseq genes that lie within the microduplicated region. These graphics were taken from the UCSC genome browser (http://genome.ucsc.edu/).

Genome-wide copy number analysis of the proband was undertaken using an Affymetrix CytoScan 750 K Array, according to the manufacturer's instructions. Regions of copy number change were determined using the Affymetrix Chromosome Analysis Suite software (ChAS) v.1.2.2 and interpreted with the aid of the UCSC genome browser (http://genome.ucsc.edu/; Human Feb. 2009 GRCh37/hg19 assembly). The array showed a female molecular karyotype with a 232 kb heterozygous interstitial deletion involving chromosome region 7q31.33 [hg19 coordinates chr7:126,531,039-126,763,294] and a 647 kb duplication involving interstitial chromosome region 17q21.31 [hg 19 coordinates chr17:43,645,879-44,292,742]. The 7q31.33 microdeletion contains part of one gene, *GRM8* (OMIM 601116), while the 17q21.31 microduplication contains a number of genes including *CRHR1* (OMIM 122561), *IMP5* (*SPPL2C*; OMIM 608284), *MAPT* (OMIM 157140), and *STH* (OMIM 607067) and a partial duplication of the *KANSL1* gene (also known as *KIAA1267*, OMIM 612452); see Figure 1.

In order to determine the genotype encompassing the *MAPT* gene, we focused on amplifying the region of intron 9 of the *MAPT* gene that carries a polymorphic 238 bp deletion, which is a characteristic marker of the H1/H2 genotypes. Fifty nanograms of genomic DNA was subjected to PCR amplification using Roche FastStart buffer (without Mg), 1.5 mM MgCl_2_, 0.4 mM dNTPs, 1 unit Roche Faststart Taq DNA polymerase, and 0.8 *μ*M of each forward and reverse primer [14]. PCR cycle conditions comprised 95°C for 5 minutes and then 35 cycles of 94°C for 30 seconds, 55°C for 30 seconds, and 72°C for 30 seconds, with a final extension at 72°C for 10 minutes. Amplified DNAs were electrophoretically separated in a 2% E-gel (Life Technologies) and the amplicons visualised under UV light. Only the 246 bp fragment was detected, suggesting the H2 genotype, hence an H2/H2/H2 haplotype.

## 4. Discussion

From the literature review, six cases of 17q21.31 microduplication syndrome have been previously reported [15–17]. Our patient together with two others also had an additional CNV. Kitsiou-Tzeli et al. [16] reported an additional 413 kb 15q11.2 deletion, and these authors have stated that this deletion may have contributed to some of the phenotypic symptoms of their patient. Patient 3 reported by Grisart et al. [17] had the common 17p11.2 deletion but was asymptomatic for NHPP (OMIM 162500) associated with this anomaly.

Molecular characterisation of our patient showed two abnormalities. In the case of the 7q31.33 microdeletion, the pathological significance of a partial deletion of the *GRM8* gene in this region has not been determined. The *GRM8* gene encodes a neurotransmitter receptor that responds to glutamate stimulation [18]. A partial duplication of the *GRM8* gene has been discovered in an individual with autistic spectrum disorder [19]. Further studies have indicated that deletions in this gene may be over-represented in some patients with ADHD [20]. While our patient does show some traits of autism, she has excellent social skills so she has not been further investigated for this condition. Therefore, we are uncertain if this partial deletion of the *GRM8* gene has a significant effect on the phenotype of our patient.

The duplicated region of 17q21.31 contains a small number of genes (Figure 1). Some are linked to a specific phenotype such as the *MAPT* gene (microtubule associated protein *TAU*). This gene encodes proteins that stabilise microtubules, which are mostly found in neurons. Loss of function abnormalities of this gene is associated with neurodegenerative disorders such as frontotemporal dementia with Parkinson's disease, progressive supranuclear palsy, and Alzheimer's disease [21, 22]. Interestingly, these disorders have also been reported to be associated with over-expression of other genes such as **α* synuclein* gene in some cases of Parkinson's disease and amyloid precursor protein (*APP* gene) in Alzheimer's disease [23, 24].

Mouse models have shown that over-expression of the murine homologue of the *TAU* gene models the impact of human H1/H1 *MAPT* haplotype, increasing the expression of the tau protein and thereby the risk of the development of associated tauopathies [25]. However, larger screening of the *MAPT* gene has shown that copy number gains have not so far been implicated in neurodegenerative diseases [26].

The *CRHR1* gene encodes a corticotrophin releasing hormone receptor type 1 and is involved in coordinating the endocrine, autonomic, behavioural, and immune responses to stress through actions in the brain and the periphery [27]. Polymorphisms in this gene have commonly been associated with depression and panic disorder [28, 29]. The *IMP5* gene encodes intramembrane protease 5, which is a part of a class of enzymes which cleave integral membrane proteins [30]. The *KANSL1* gene encodes a nuclear protein which plays a role in chromatin modification. It encodes two subunits which play a role in a histone acetyltransferase complex [31]. Finally, the *STH* gene is a polymorphic gene nested within an intron of the *MAPT* gene and encodes the protein Saitohin [32], the function of which is not yet known.

Table 1 summarises the clinical phenotypes of the limited number of 17q21.31 microduplication cases reported thus far. In most cases pregnancies tend to be very uneventful (5/7) and early milestones normal (7/7). One of the first main signs has been hypotonia (3/7 cases), with tiptoe walking (3/7 cases) and microcephaly (3/7). Verbal skills vary widely from normal to very poor, while motor skills also appear to vary in the number of cases (3/7). Dysmorphic features have in general not been noticed until later in life and are often quite subtle, while some dysmorphic features like clinodactyly, syndactyly (2/7), and hypogonadism (1/7) have been reported infrequently. Both our patient and patient 3 reported by Grisart et al. [17] do not appear to suffer any increased phenotypic severity despite the presence of an additional CNV.

In contrast to the above, the corresponding 17q21.31 microdeletion syndrome is characterised by developmental delay, hypotonia, facial dysmorphisms, and a friendly, amiable behaviour [33–35]. A number of recent reports have shown that loss of function of the *KANSL1* gene within this region is sufficient to cause the syndrome [12, 13]. The 17q21.31 microduplication phenotype appears milder, while some of the more severe congenital malformations seen in the microdeletion syndrome such as urological anomalies, ventriculomegaly, musculoskeletal problems, dental abnormalities, and epilepsy have not been reported in patients with the corresponding microduplication [33–35]. Also of interest is a recent report of a *de novo* triplication of the *MAPT* and *KANSL1* genes which extends into the polymorphic region of 17q21.31 [36]. The phenotype of this patient included behavioural and social problems, muscular hypotonia, hypoplastic genitalia, cryptorchidism, clinodactyly, and mild facial dysmorphisms, which is largely similar to the microduplication cases with minor facial features, behavioural problems, and moderate mental impairment.

The extent of the 17q21.32 microduplication among the patients shown in Table 1 varies from approximately 239 kb to 736 kb. An assessment of published cases and information from the DECIPHER database (http://decipher.sanger.ac.uk/) suggests that none of the cases contained full duplications of all of the main genes contained within the *MAPT* haplotype. Our patient contained a full copy of *MAPT *and *CRHR1* and a partial copy of *KANSL1* genes. Two patients reported by Kitsiou-Tzeli et al. [16] and Grisart et al. [17] (patients 4, Figure 1) appear to be the only cases reported thus far that do not contain a full or partial duplication of *CRHR1* gene. Our patient has a similar duplication to that of patient 1 reported by Grisart et al. [17] who has a 646 kb deletion, but with slightly varying breakpoints and with only a partial duplication of *CRHR1*. Both phenotypes were similar with common features including tiptoe walking, microcephaly, and poor motor skills. The main difference between these two cases appeared to be hypotonia and more severe dysmorphic features. Patient 4 reported by Grisart et al. [17] carries the smallest microduplication reported so far of 0.24 Mb. It contains a full duplication of the *MAPT* gene and a very small partial duplication of the *KANSL1* gene. This patient's phenotype appears to be the mildest of the cases reported with normal verbal and motor skills, psychomotor development and retardation similar to other cases, and mild dysmorphic features. Interestingly, the patient reported by Kirchhoff et al. [15] appears to lack a duplication of the *KANSL1 *gene but has a full duplication of the *MAPT* and *CRHR1* genes and manifests similar psychomotor development, more severe psychomotor retardation, poorer verbal skills, and microcephaly in comparison to this case. A degree of variable penetrance may exist within the phenotype of the cases reported so far. Therefore while we cannot rule out the contribution of any gene or part of gene in the haplotype to the overall phenotype, patient 4 reported by Grisart et al. [17] may represent the minimum duplication of the 17q21.31 region that characterises this microduplication syndrome. It is possible that a minimum critical region encompassing the *MAPT* gene may emerge when further cases become apparent.

The *MAPT* gene is flanked by LCRs (Figure 1). NAHR involving LCRs can lead to deletion and duplication events, and the complex structure of LCRs that comprise both direct and indirect repeat elements can lead to inversions [5–8, 37]. While still to be determined for humans, *in vivo* experiments using mouse cells have shown that a minimum efficient processing segment (MEP) between 134 and 232 bp of perfect shared sequence identity is required for homologous recombination [3]. Koolen et al. [33] have identified an approximate 500 bp within an L2 LINE repetitive element motif at 17q21.31, representing a possible proximal hotspot for NAHR within this haplotype. The distal breakpoint in the 17q21.31 microdeletion syndrome cases has been determined to be more variable due to it being located in a polymorphic region adjacent to the critical deletion region [33].

Compared to the corresponding microdeletion syndrome, 17q21.31 microduplications have been seen less frequently. A recent study [15] reported the frequency among live births of 1/55,000 and 1/327,000 for microdeletions and microduplications, respectively, which suggests a ratio of 6 : 1, which is lower than expected of NAHR [6]. Importantly, these ratios should be viewed against the molecular background in which they occur and the genomic region being investigated in that the H2 inversion haplotype is expected to favour microdeletions as opposed to microduplications [11]. The case reported here carries the H2 haplotype but is associated with a copy number gain rather than loss. Finally, it is possible that cases of 17q21.31 microduplication syndrome have been underascertained due to the milder phenotype and later onset.

## Figures and Tables

**Table 1:** Comparison of main clinical indications reported on seven patients with 17q21.31 microduplications.

Reference	Present case	Kirchhoff et al. [15]	Kitsiou-Tzeli et al. [16] Patient 4	Grisart et al. [17] Patient 1	Grisart et al. [17] Patient 2	Grisart et al. [17] Patient 3	Grisart et al. [17] Patient 4
hg19 coordinates	43,645,879–44,292,742	43,675,408–44,159,862	43,933,055–44,628,150	43,717,703–44,345,038	43,778,601–44,364,056	~43,744,217–~44,344,223	43,932,635–44,172,437
Pregnancy	38 weeks, suspected IUGR	Uneventful 42 weeks	40 weeks	Placental detachment at term	Normal at term	Normal at term	Normal at term
Birth (gms)	2.490	3.070	2500	3.570 (P50)	3.500 (P50)	2.890 (22SD)	5.100 (+3SD)
Walk (months)	15	60	13	12	24	14–16	27
Tiptoe walking	+	ND	ND	+	−	+	−
Psychomotor Development							
Hypotonia	ND	+	−	−	+	−	+
Hyperactivity	ND	+	ND	+	−	ND	+
Passivity	ND	ND	ND	ND	+	ND	ND
Obsessive behaviour	ND	ND	ND	ND	+	ND	−
Social interaction	ND	ND	Outburst of temper	Poor	Poor	Poor, anxious	Poor
Intelligence							
Psychomotor retardation	ND	Severe	ND	Mild	Mild	Mild	Mild
Verbal skills	Normal	Very poor	ND	Limited	Normal	Poor	Normal
Motor skills	Poor	ND	ND	Poor	Poor	ND	Normal
Dysmorphism							
Microcephaly	+	+	ND	Mild	−	−	−
Synophrys	ND	ND	ND	+	Bushy eyebrows	−	−
Epicanthic folds	ND	ND	ND	−	+	−	−
Dysplastic ears	ND	+	ND	+	+	−	+
Nose	ND	Short	Short nose, prominent nasal tip, and columella	Upturned	Short, upturned	−	−
Philtrum	ND	Smooth	Smooth	Short	Short	−	−
Midface	ND	ND	Small mouth	−	Flat	−	Flat
Palate	ND	High arched	ND	−	High arched	−	−
Prominent incisor	ND	+	ND	+	+	−	−
Micrognathia	ND	+	Mild	ND	−	ND	−
Finger	ND	Broad	ND	Clinodactyly of 5th	Tapering	ND	Clinodactyly of 5th
Palmar crease	ND	ND	ND	Single on right hand	−	ND	ND
Feet	ND	Broad	ND	Partial syndactyly	−	ND	2-3 Syndactyly
Others							
	Almond shaped eyes, small hands	Hirsutism on back	Global hirsutism, ataxic gait, VSD	Hirsuitism on back	Low posthairline		Hypogonadism

ND: not determined, +/−: trait present/not present in patient.
